# Supplementary material for: β-COP Suppresses the Surface Expression of the TREK2
Source: Cells. 2023 May 29;12(11):1500. doi: 10.3390/cells12111500 (PMC10252889; doi:10.3390/cells12111500)
Supplement: Supplementary file 1 [file cells-12-01500-s001.zip › cells-2364726-supplementary.pdf]

Supplementary Figure 1. Alignment of N- and C- terminus of TREK2 and TREK1

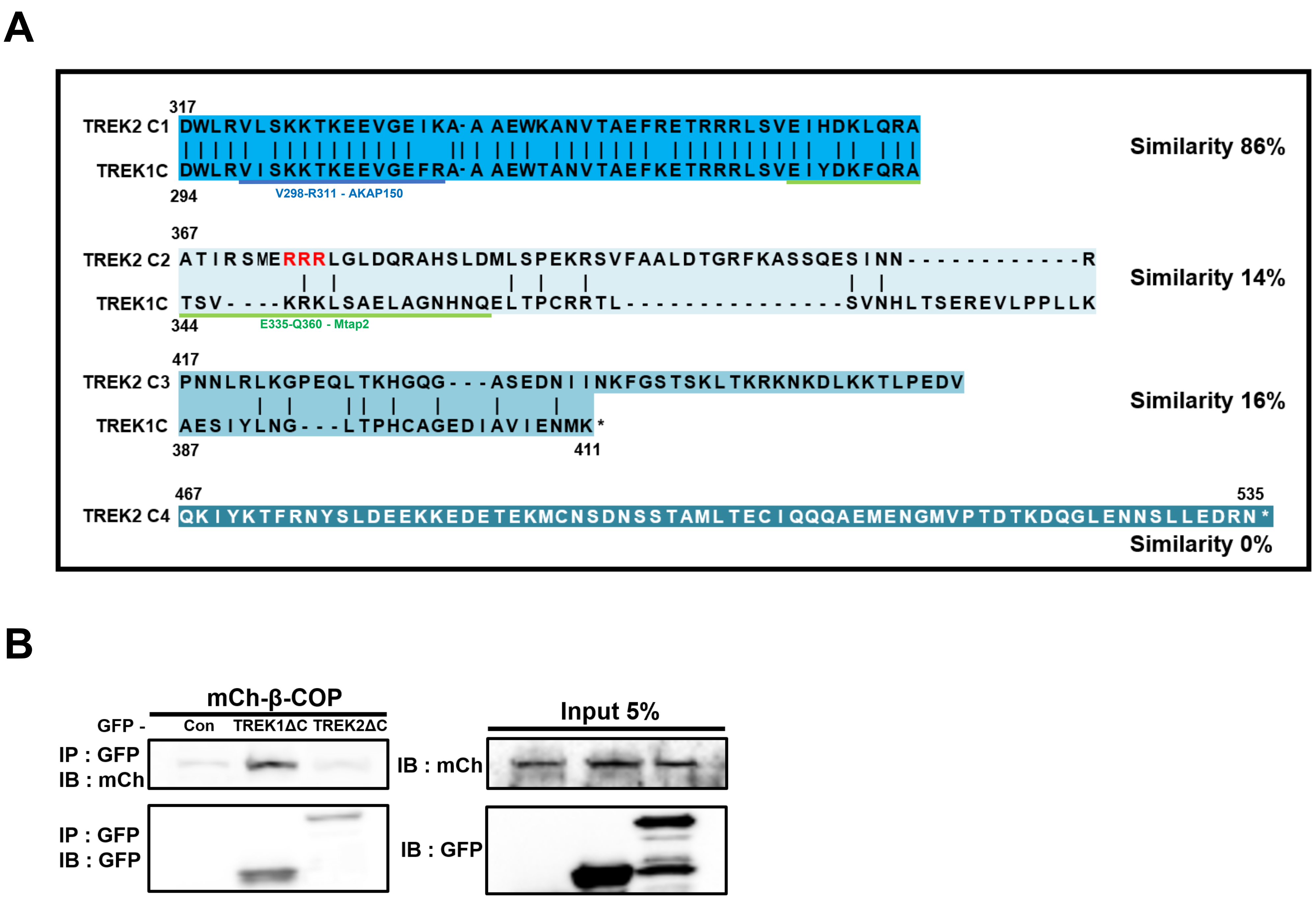

Supplementary Figure 1. Alignment of N- and C- terminus of TREK2 and TREK1.
(A) Comparative alignment of the C-terminus of TREK2 divided into quarters and the corresponding C-terminus of TREK1. The degree of similarity was calculated as the ratio of identical amino acids in TREK1 and TREK2 to all the amino acids in each region. (B) Co-immunoprecipitation of HEK293T cells transfected with GFP-tagged TREK1ΔC, TREK2ΔC, and mCh-β-COP. Immunoprecipitation was performed using green fluorescent with GFP and blotted with RFP.
